# Neuromorphic chip integrated with a large-scale integration circuit and amorphous-metal-oxide semiconductor thin-film synapse devices

**DOI:** 10.1038/s41598-022-09443-y

**Published:** 2022-03-30

**Authors:** Mutsumi Kimura, Yuki Shibayama, Yasuhiko Nakashima

**Affiliations:** 1 Graduate School of Science and Technology, Nara Institute of Science and Technology (NAIST), Takayama, Ikoma, 630-0192 Japan; 2 Graduate School of Science and Technology, Ryukoku University, Seta, Otsu, 520-2194 Japan

**Keywords:** Electrical and electronic engineering, Electronic devices, Computer science

## Abstract

Artificial intelligences are promising in future societies, and neural networks are typical technologies with the advantages such as self-organization, self-learning, parallel distributed computing, and fault tolerance, but their size and power consumption are large. Neuromorphic systems are biomimetic systems from the hardware level, with the same advantages as living brains, especially compact size, low power, and robust operation, but some well-known ones are non-optimized systems, so the above benefits are only partially gained, for example, machine learning is processed elsewhere to download fixed parameters. To solve these problems, we are researching neuromorphic systems from various viewpoints. In this study, a neuromorphic chip integrated with a large-scale integration circuit (LSI) and amorphous-metal-oxide semiconductor (AOS) thin-film synapse devices has been developed. The neuron elements are digital circuit, which are made in an LSI, and the synapse devices are analog devices, which are made of the AOS thin film and directly integrated on the LSI. This is the world's first hybrid chip where neuron elements and synapse devices of different functional semiconductors are integrated, and local autonomous learning is utilized, which becomes possible because the AOS thin film can be deposited without heat treatment and there is no damage to the underneath layer, and has all advantages of neuromorphic systems.

## Introduction

Artificial intelligences are used for a variety of purposes and are promising in future societies^1^. Neural networks are typical technologies, and its advantages include self-organization, self-learning, parallel distributed computing, and fault tolerance^2^. However, the conventional ones are complicated software on high-spec hardware, so their size and power consumption are large^3^. In addition, they run on von Neumann computers^4^, so they do not offer some benefits. Neuromorphic systems are biomimetic systems from the hardware level, with the same advantages as living brains, especially compact size, low power, and robust operation^5^. However, while some neuromorphic systems are well known^6^, they are non-optimized systems that use conventional digital technology, so the above benefits are only partially gained. In particular, machine learning is processed on server computers and only fixed parameters are downloaded to the neuromorphic systems^7^. Incidentally, while recent neuromorphic systems are excellently sophisticated^8–11^, they still use digital technology and have the above benefits partially.

We are researching neuromorphic systems. To date, we have reported the research results of actual experiments on amorphous-metal-oxide semiconductor (AOS) thin-film binary and analog memristors^12–15^, AOS thin-film spike-timing-dependent-plasticity (STDP) devices^16,17^, and a neuromorphic system equipped with crossbar-type AOS thin-film synapses^18^, and the simulation results on a neuromorphic chip integrated with a large-scale integration circuit (LSI) and AOS thin-film synapse devices^19^, together with local autonomous learning^20^. In this research, as a culmination of the above research results, we have developed a neuromorphic chip integrated with an LSI and amorphous-metal-oxide semiconductor (AOS) thin-film synapse devices utilizing local autonomous learning. In this article, we will explain the structure of devices and systems, operating principle, and operation confirmation as an associative memory. Our neuromorphic chip is the world's first hybrid chip in which neuron elements made in an LSI and synapse devices made of different functional semiconductors are integrated on the same wafer, and local autonomous learning is utilized. Compared to previous reports of similar studies^21,22^, the LSI and AOS thin-film synapse devices are integrated on the same wafer, which becomes possible because the AOS thin film can be deposited without heat treatment, and there is no damage to the underneath layer. Moreover, compared to other previous reports^23,24^, the local autonomous learning is utilized, which becomes possible because required properties can be added by controlling the materials, devices, and processes. As a result, our neuromorphic chip has the potential to have all the above advantages of neuromorphic systems.

## Results

### Neuron elements made in an LSI

**Figure 1:**
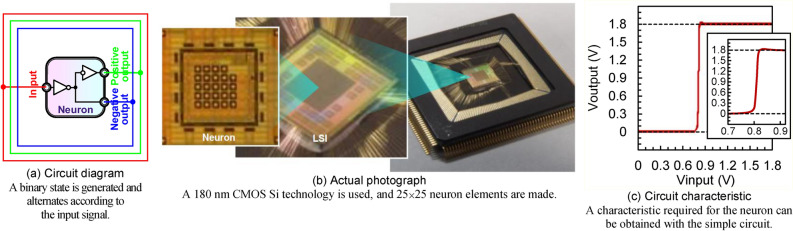
Neuron elements made in an LSI.

Figure 1 shows the neuron element made in an LSI. Figure 1a shows the circuit diagram. The neuron element is a digital circuit consisting of four transistors with two inverters connected in series. A binary state, that is, stable or firing state, is generated and alternates according to the input signal. The input, positive output, and negative output terminals are unidirectional. If the input signal is above the threshold voltage of the inverter, the stable state is generated, the positive output signal becomes Vss, and the negative output signal becomes complementarily Vdd. On the other hand, if the input signal is below the threshold voltage, the firing state is generated, the positive output signal becomes Vdd, and the negative output signal becomes complementarily Vss. The theoretical model of the neuron element is just a buffer block, which has exactly the same function as a two-inverter circuit. Figure 1b shows the actual photograph. A 180 nm CMOS Si technology is used^25^, 25 × 25 neuron elements are made in the LSI, 12 × 12 neuron elements are accessible every other column and row, these are used as input/output (I/O) neurons to see if the neuromorphic system works, and hidden neuronal elements are located between I/O neurons. Since the LSI is common digital circuits, they can be easily manufactured using the traditional method of Si CMOS FETs. Before the AOS thin-film synapse devices are integrated, the neuron elements are isolated and not connected anywhere. Figure 1c shows the circuit characteristic. The horizontal axis is Vinput, input signal actually applied to the input terminal, while the vertical axis is Voutput, positive output signal actually measured from the positive output terminal shown in Fig. 1a. Vss is 0 V, and Vdd is 1.8 V. It is found that a step function or slight sigmoid function can be obtained, which are representative functions required for the neuron element. It turns out that a circuit characteristic required for the neuron element can be obtained with the simple circuit.

### Synapse device made of the AOS thin film

**Figure 2:**
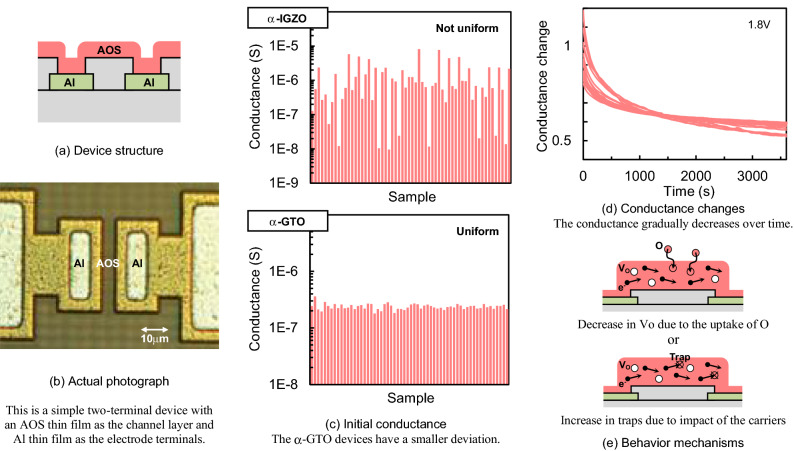
Synapse device made of the AOS thin film.

Figure 2 shows the synapse device made of the AOS thin film. The synapse device is an analog device whose conductance continuously changes. Figure 2a shows the device structure, and Fig. 2b shows the actual photograph. This is a simple two-terminal device, with an AOS thin film used as the channel layer and Al thin film as the electrode terminals. The channel width corresponds to the width of the contact holes on the electrode terminals and is 20 µm, whereas the channel length corresponds to the distance between the contact holes and is 15 µm.

Figure 2c shows the initial conductance. The synapse device made of the amorphous In-Ga-Zn-O thin film (α-IGZO) and that made of the amorphous Ga-Sn–O thin film (α-GTO) are compared. The α-IGZO is deposited using radio-frequency (RF) magnetron sputtering, where the sputtering target is an IGZO ceramic with a composition of In:Ga:Zn = 1:1:1, the sputtering gas is Ar with a flow rate of 20 sccm, the deposition pressure is 5 Pa, the plasma power is 60 W, the deposition time is 25 min, etc. The α-GTO is also deposited using RF magnetron sputtering, where the sputtering target is a GTO ceramic with a composition of Ga:Sn = 1:3, etc., and the thickness is 100 nm. Both have not undergone a heat treatment. It turns out that the synapse devices made of the α-IGZO have a larger deviation in initial conductance than that made of the α-GTO. This seems to be due to the fact that the α-IGZO is a quaternary system and has a considerable variation in the element ratio from sample to sample. Furthermore, Zn is chemically active, and it is difficult to control the chemical condition uniformly without the heat treatment. The α-GTO is a ternary system, and the variation is small without the heat treatment. Moreover, since the α-GTO does not contain rare metals like In, there is almost no risk of resource depletion or cost increase, which is extremely useful in applications that uses large amounts of materials, such as synapse devices in neuromorphic systems. Therefore, the α-GTO is used in this research.

Figure 2d shows the conductance changes. The changes of the conductance are measured when a voltage of 1.8 V is applied to the synapse devices. It turns out that the conductance gradually decreases over time. Figure 2e shows the behavior mechanisms. One is the decrease in oxygen vacancies due to the uptake of oxygen, and the other is the increase in traps due to impact of the carriers. In any case, the conductance changes are used as a local autonomous learning in this research.

### Local autonomous learning

**Figure 3:**
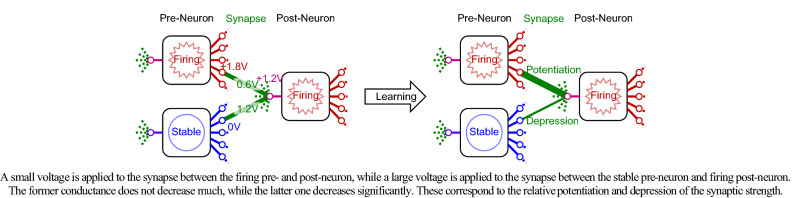
Local autonomous learning.

Figure 3 shows the local autonomous learning. The neuron elements are directly connected through the synapse devices. In this example, it is assumed that one pre-neuron is in a firing state and the output voltage is 1.8 V, the other pre-neuron is in the stable state and the output voltage is 0 V, and the post-neuron is in the firing state and the input voltage is 1.2 V, which is above the threshold voltage. As a result, a small voltage of 0.6 V is applied to the synapse device between the pre-neuron and post-neuron in the firing state, while a large voltage of 1.2 V is applied to the synapse device between the pre-neuron in the stable state and the post-neuron in the firing state. The conductance of the former synapse device does not decrease much, while that of the latter one decreases significantly. These correspond to the relative potentiation of the synaptic strength of the former one and the relative depression of the latter one. This local autonomous learning rule is a modified version of Hebbian learning rule and its operation has been confirmed by logical simulation^15^, where the conductance change of the AOS thin-film synapse device was modeled, the local autonomous learning rule was used, and the operation of a neuromorphic system was confirmed. This local autonomous learning rule is used in this research.

### Neuromorphic chip integrated with an LSI and AOS thin-film synapse devices

**Figure 4:**
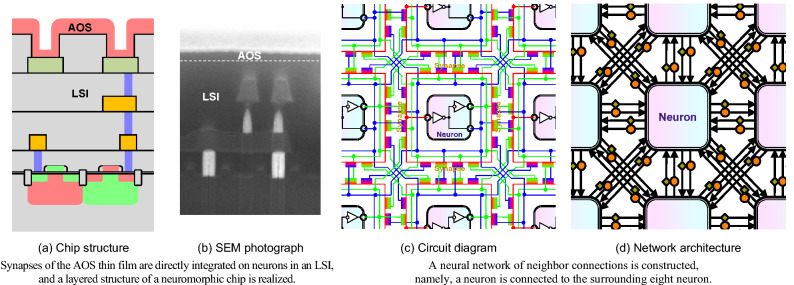
Neuromorphic chip integrated with an LSI and AOS thin-film synapse devices.

Figure 4 shows the neuromorphic chip integrated with an LSI and AOS thin-film synapse devices. Figure 4a shows the chip structure, and Fig. 4b shows the cross-sectional scanning-electron-microscope (SEM) photograph. The above-mentioned synapse devices made of the AOS thin film are directly integrated on the above-mentioned neuron elements made in an LSI, and the layered structure of the neuromorphic chip is realized. Figure 4c shows the circuit diagram, and Fig. 4d shows the corresponding network architecture. A neural network of neighbor connections is constructed, namely, a neuron element is connected to the surrounding eight neuron elements, that is, four orthogonal front, back, right, and left, and four diagonal neuron elements, through the synapse devices. Moreover, the concordant synapses indicated by the orange color are connected to the same sign logics, namely, the positive output of the pre-neuron and input of the post-neurons, and try to make the state in the post-neuron the same as that in the pre-neuron, while the discordant synapses indicated by the green color are connected to the different sign logics, namely, the negative output and input, and try to make the state in the post-neuron different from that in the pre-neuron. In summary, a neuron element is connected to the surrounding eight neuron elements through the sixteen synapse devices, half of which are the concordant synapses, and the rest are the discordant synapses, which is also our new idea.

### Evaluation method and results as the associative memory

**Figure 5:**
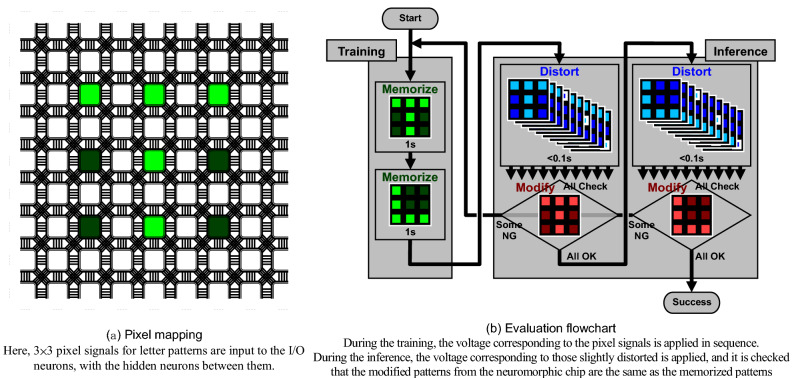
Evaluation method as the associative memory.

Figure 5 shows the evaluation method as the associative memory. Letter recognition is adopted as a benchmark test. Figure 5a shows the pixel mapping. Here, 3 × 3 pixel signals for letter patterns are input to the I/O neuron elements, with the hidden neuron elements placed between them. Figure 5b shows the evaluation flowchart. During the training phase, the voltage corresponding to the pixel signals for the two letter patterns of “T” and “L” is applied in sequence. Since the voltage is applied for as long as 1 s, the conductance changes in the synapse devices. During the inference phase, the voltage corresponding to those slightly distorted from “T” and “L” is applied in sequence. Since the voltage is applied for a short period less than 0.1 s, the conductance does not change. After a while, it is checked that the modified patterns returned from the neuromorphic chip are the same as the memorized patterns. If at least one of the modified patterns is different from the memorized pattern, this flowchart is repeated.

**Figure 6:**
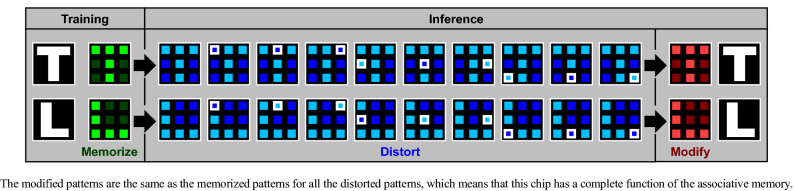
Evaluation results as the associative memory.

**Figure 7:**
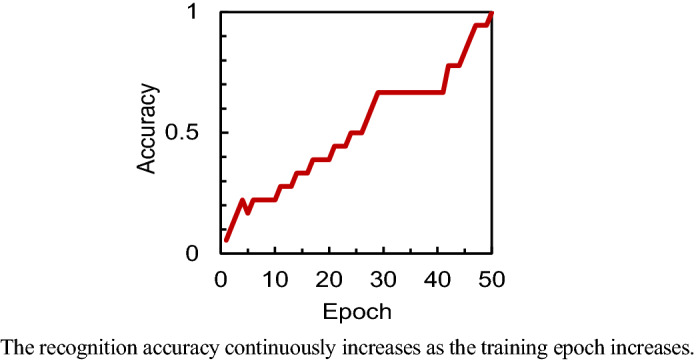
Training epoch and recognition accuracy.

Figure 6 shows the evaluation results as the associative memory. The flowchart of the training and inference phases is repeated dozens of times. It turns out that the modified patterns are the same as the memorized patterns for all the distorted patterns, which means that this chip has a complete function of the associative memory. Figure 7 shows the training epoch and recognition accuracy. The training epoch is defined as the number of repeats of the flowchart shown in Fig. 5b, and the recognition accuracy is defined as the rate of the distorted patterns that are correctly modified to the memorized patterns. It is found that the recognition accuracy continuously increases as the training epoch increases, which is owing to that the conductance of the synapse device sufficiently slowly decreases as shown in Fig. 2d. Moreover, the recognition accuracy reaches 1 when the training epoch is 50. Incidentally, although the demonstrated performance of the neuromorphic chip is limited to the associative memory in this article, because it is a typical application of artificial intelligences, various applications can be expected in the future.

## Conclusion

In conclusion, a neuromorphic chip integrated with an LSI and AOS thin-film synapse devices utilizing local autonomous learning has been developed. The neuron elements are digital circuit, which were made in an LSI, and the synapse devices are analog devices, which were made of the AOS thin film and directly integrated on the LSI. It turned out that this chip has a complete function of the associative memory, which is a typical application of artificial intelligences. This is the world's first hybrid chip where neuron elements and synapse devices of different functional semiconductors are integrated, and local autonomous learning is utilized, and has all advantages of neuromorphic systems.

## Methods

For the readers to easily understand the content, the constituent materials, device structures, circuit configurations, element designs, and fabrication processes of the neuron elements, synapse devices, and neuromorphic chips have been already explained above. The operation principle of the local autonomous learning has been also described above in detail. The Evaluation method as the associative memory has been also already written above.
